# Implementing technology in healthcare: insights from physicians

**DOI:** 10.1186/s12911-017-0489-2

**Published:** 2017-06-27

**Authors:** Maria Dolors Ruiz Morilla, Mireia Sans, Albert Casasa, Nuria Giménez

**Affiliations:** 1 CAP Terrassa Oest. MútuaTerrassa, Terrassa, Barcelona, Spain; 2 Health 2.0 section of the Col·legi Oficial de Metges de Barcelona, Barcelona, Spain; 3 CAP Comte Borrell. CAPSBE. Hospital Cínic, Barcelona, Spain; 4 EAP Sardenya. Biomedical Research Institut Sant Pau, Barcelona, Spain; 5 Mútua Terrassa Research Foundation, Universitat de Barcelona, Barcelona, Spain; 6 Toxicology Laboratory, Universitat Autònoma de Barcelona, Barcelona, Spain

**Keywords:** Attitude to health, Health knowledge, Attitudes, Telemedicine, Delivery of healthcare, Diffusion of innovation, Physician-patient relations, Attitude to computers, Surveys and questionnaires, Organizational innovation

## Abstract

**Background:**

Technology has significantly changed the way health organizations operate. However, the role it plays in healthcare systems remains unclear. This aim of this study was to evaluate the opinion of physicians regarding e-health and determine what factors influence their opinion and describe the advantages, inconveniences and threats they may perceive by its use.

**Methods:**

A cross-sectional questionnaire-based study. A questionnaire which had been previously designed and validated by the authors was used to interview physicians from the Barcelona Medical Association. 930 physicians were contacted by phone to participate in the study.

**Results:**

Seven hundred sixty physicians responded to the questionnaire (response rate: 82%). The usefulness of telemedicine scored 7.4 (SD 1.8) on a scale from 1–10 (from the lowest to the highest) and the importance of the Internet in the workplace was 8.2 points (SD 1.8). Therapeutic compliance (7.0 -SD 1.8-) and patient health (7.0 -SD 1.7-) showed the best scores, and there were differences between professionals who had and had not previously participated in a telemedicine project (*p* < 0.05). The multivariate regression model explained the 41% of the variance for 7 factors: participation in telemedicine project (*p* < 0.001), quality of clinical practice (*p* < 0.001), patient health (*p* < 0.001), professional workload (*p* = 0.005), ease-of-use of electronic device (*p* = 0.007), presence of incentives for telemedicine (*p* = 0.011) and patient preference for in-person visits (*p* = 0.05).

**Conclusions:**

Physicians believe in the usefulness of e-health. Professionals with previous experience with it are more open to its implementation and consider that the benefits of technology outweigh its possible difficulties and shortcomings. Physicians demanded projects with appropriate funding and technology, as well as specific training to improve their technological abilities. The relationship of users with technology differs according to their personal or professional life. Although a 2.0 philosophy has been incorporated into many aspects of our lives, healthcare systems still have a long way to go in order to adapt to this new understanding of the relationship between patients and their health.

**Electronic supplementary material:**

The online version of this article (doi:10.1186/s12911-017-0489-2) contains supplementary material, which is available to authorized users.

## Background

There has been a rise in the demand in healthcare systems in Western countries due to aging of the population, an increase in the prevalence of chronic diseases, and limitations in funding, especially after the recent economic recession [1]. In this context, the introduction of computers and technology can help to improve the efficiency of the healthcare system and the care provided to patients/users [2].

For the purpose of this paper we use the terminology in the following way. *E –health* is used referring to health services and information delivered or enhanced through the Internet and related technologies [3]. We consider *telemedicine* (TM) as the use of medical information to improve the health of patients via electronic communication [4]. E-health allows access to health resources and healthcare by electronic means [5]. It provides an opportunity to not only to preserve or improve the quality of healthcare more cost-effectively but also allows healthcare services to be reinvented in order to make them more dynamic and able to adapt to technological changes. Finally we talk about having a 2.0 attitude in relation to having incorporated the principles of Web 2.0. It is characterized by greater user interactivity and collaboration, more pervasive network connectivity and enhanced communication channels.

The role of TM in public healthcare is controversial. Many technological solutions are currently possible [6], however, it is not the technology of TM that is important but rather the new approach to provide and organize healthcare services. Indeed, TM changes physician-patient relationships allowing more direct patient involvement in the decision making related to their health [7–9].

However, despite political commitment and significant investment, the application of technology to healthcare systems has not always been successful [4, 10]. The factors which can facilitate or hinder the introduction of TM in healthcare have been described in depth [11], with legal and regulatory issues, questions involving reimbursement and the impact on the effectiveness and the quality of care being the most common barriers reported [6, 12].

The success of TM depends on the end-users, that is the physicians and patients actually using it, and this largely depends on how it is implemented [13]. Different models have been proposed to predict what factors will determine its success. For example, the Technology Acceptance Model (TAM) has been applied to determine how physicians come to accept and use TM [14], and two factors have been identified as important predictors of the use of technology: perceived ease-of-use and perceived usefulness [15–17].

Thus, physicians must be involved and their needs taken into account in order to implement this change [18]. Moreover, it is important to understand the relationship between physicians and technology and how they evaluate the introduction of new technologies in their daily clinical practice.

Taking all of the above into account, the main objective of this study was to evaluate the opinions of physicians regarding e-health. Secondary objectives were to evaluate what variables influence their opinion regarding e-health, to describe the advantages, inconveniences and threats these professionals perceive with the use of telemedicine and how they use new technologies.

## Methods

A cross-sectional questionnaire-based study was designed. A TM questionnaire which had previously been designed and validated by our team in collaboration with other healthcare professionals was used [19]. A new section on the use of technological devices was added to the questionnaire. In total, the questionnaire included eight theoretical sections and 46 variables (Additional file 1: Table S1).

**Fig. 1:**
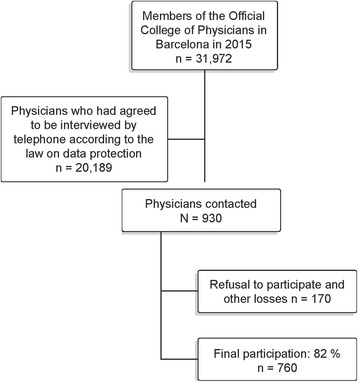
Participation diagram

The study population consisted of physicians belonging to the Barcelona Medical Association -*Col · legi Oficial de Metges de Barcelona*- (*n* = 31,972) the official institution including all the physicians practicing in the province of Barcelona (Spain). Only physicians who had agreed to be interviewed by telephone were chosen (*n* = 20,189) according to the law on data protection and were randomized. A total of 930 physicians were contacted by phone from May to June 2015, 760 of whom responded to the questionnaire (response rate 82%). A researcher recorded their response to each question done over the phone. Participation was voluntary, and information on the nature of the study was provided during the telephone call. The participants were informed that the information would only be used for the purpose of investigation and confidentiality and anonymity were guaranteed. Details on participation are provided in Fig. 1.

According to Murray et al., we consider professionals who are dedicated to management to be “implementers” because of their role in the planning of healthcare services and in the final decision as to whether or not to incorporate technology into these services [20]*.*

The professionals studied were classified into three age groups: under 40 years of age, 40–50 years, and over 50 years old.

### Statistical analysis

We evaluated the reliability of the questionnaire using the Cronbach alpha coefficient. No variable presented losses greater than 5%. In addition, quantitative variables were expressed as numbers and percentages, and semi-quantitative variables were scored on a scale of 1–10 (from the lowest to the highest) and were expressed quantitatively with at least one measurement of central tendency and one of dispersion. The normality was explored with the Kolmogorov-Smirnov test. The Chi-square test was used to compare qualitative variables and means were compared with the Student’s *t* test and ANOVA. In the multivariate multiple regression model, the evaluation of the utility of TM by the physicians was considered as a dependent variable, and predicted variables were those showing statistical significance in the bivariate analysis adjusted for age. The enter method was used to perform the analyses. Statistical significance was accepted with a *p* value < 0.05, with a bilateral approach. The SPSS 17.0 programwas used (SPSS Inc., Chicago, USA).

### Data exclusion

Participants who refused to participate in the study were excluded, as were those who could not be contacted.

## Results

Seven hundred sixty physicians responded to our survey, being a response rate of 82% (Fig. 1).

All of the questions were answered by more than 95% of participants.

Table 1 shows the characteristics of the study population; 53% were women with a mean age of 46 years (SD 11).

**Table 1:** Socio-demographic data of the physicians included in the study

Variable	Frequency (percentage)
N total	760 (100%)
Age	
Under 40	285 (38%)
41–50	163 (21%)
Over 50	312 (41%)
Sex	
Male	355 (47%)
Female	405 (53%)
Residence	
Barcelona area	444 (58%)
Other	316 (42%)
Specialty	
Primary	348 (46%)
Hospital:	
Medical	240(32%)
Surgical	104 (14%)
Central support services	68 (9%)
Sector	
Public	425 (56%)
Private	142 (19%)
Both	188 (25%)
Place of work	
Primary health centre	312 (41%)
Level 3 hospital	169 (22%)
Level 1–2 hospital	112 (15%)
Private practice	112 (15%)
Healthcare centre	25 (3%)
Other	30 (4%)
Position	
Medical staff	697 (92%)
Management	61 (8%)

### Opinion of e-health

#### Participation in telemedicine and the perception of its usefulness

The mean score of the usefulness of TM was 7.4 (SD 1.8). Thirty percent of the responders reported having previously participated in a TM project. This participation was significantly more frequent among physicians in the public compared to the private sector (*p* < 0.001), especially among physicians in primary care or third level hospitals.

Table 2 describes the factors that influence the usefulness of TM. Physicians with previous experience with TM scored its utility higher and perceived greater possibilities or benefits with its use. No differences were found in the area of needs and difficulties.

**Table 2:** Factors that influence the usefulness of TM

Opportunities –benefits			Needs		Difficulties	
Item	Average score ± SD		Item	Average score ± SD	Item	Average score ± SD
	Has experience with TM	Has not experience with TM				
Frequency of in-person visits	6.5 ± 2.0	6.2 ± 1.9	Patients’ preference for in-person visits	6.5 ± 2.1	Safety and confidentiality of information	5.7 ± 2.3
Administrative work	6.4 ± 2.1	6.3 ± 2.2	Professionals’ preference for in-person visits	6.4 ± 2.0	Ease-of-use of electronic devices	6.9 ± 1.9
Therapeutic compliance*	7.0 ± 1.8	6.5 ± 1.8	Patients’ technological skills	6.7 ± 1.9	Record of profesionals’ performance	6.8 ± 2.0
Healthcare costs	6.7 ± 1.7	6.5 ± 1.8	Professionals’ technological skills	6.9 ± 1.9	Needfor training	7.1 ± 1.9
Quality of clinical practice*	6.9 ± 1.9	6.4 ± 1.9	Appropiate electronic device	7.6 ± 2.0	Technical difficulties in the use of TM	6.3 ± 2.0
Patient health*	7. 0 ± 1.7	6.4 ± 1.6	Project funding	7.8 ± 2.0	Time needed for electronic visits	6.4 ± 2.1
Professional workload	6.1 ± 2.1	6.1 ± 2.1	Time needed for each patient	6.2 ± 2.0	Presence of incentives for TM	6.2 ± 2.3
Sum of opportunities*	6.6 ± 1.2	6.3 ± 1.3	Sum of needs	6.9 ± 1.2	Sum of difficulties	6.5 ± 1.2

Variables are scored on a scale of 1–10 (from least to greatest)

*p < 0.05 difference between having or not having previous experience in TM project using the T-student test

Table 3 shows the data from the multiple regression model with the usefulness of TM as a dependent variable. The independent variables were those found to be significant in the bivariate analysis in addition to age. The factors that were found to influence telemedicine were previous experience with telemedicine, the quality of clinical practice and patient health.

**Table 3:** Multivariate model. Factors that influence the usefulness of telemedicine, adjusted for age. R2 = 0.41%

Variable	B(CI 95%)	*p*-value
-Experience with telemedicine -Quality of clinical practice -Patient health -Professional workload -Ease-of-use of electronic device -Presence of incentives for telemedicine -Patients’ preference for in-person visits	0.53(0.32–0.76) 0.25(0.18–0.32) 0.34(0.26–0.42) 0.07(0.02–0.12) 0.08(0.02–0.14) 0.06(0.01–0.10) 0.05(0.01–0.10)	<0.001 <0.001 <0.001 0.005 0.007 0.011 0.05

#### Differences according to age and devices available

Professionals under the age of 40 years with a smartphone scored the utility of TM higher than those without and considered TM to be able to improve the health of patients and therapeutic compliance (Additional file 2: Table S2).

No differences were observed in the opinion of professionals regarding TM according to whether or not they had a PC.

Physicians over the age of 50 with a tablet gave more importance to the Internet in the workplace and evaluated the usefulness of TM higher compared to those without a tablet. These physicians also considered that TM would improve the quality of clinical practice, patient health, therapeutic compliance, healthcare costs and administrative work. This group expected more difficulties in the introduction of TM into clinical practice and were more concerned about the ease-of-use of the devices, registry of professional performance and the presence of incentives. They also considered that adequate funding was essential.

#### Implementers and telemedicine

Physicians working in management scored the following areas higher compared to the remaining participants: benefits of TM in therapeutic compliance (7.2 vs. 6.6, *p* = 0.02), improvement of healthcare costs (7.1 vs. 6.5, *p* < 0.01), and administrative work (7.1 vs. 6.2, *p* < 0.01), and the need for incentives for professionals to use TM to ensure its success (6.8 vs. 6.2, *p* < 0.06).

#### Private medicine and the perception of telemedicine

Physicians working in private medicine scored the following areas higher than those in public institutions: benefits of TM in improving patient health (6.8 vs. 6.5, *p* <0.05), therapeutic compliance (7.0 vs. 6.5, *p* < 0.04), professional workload (6.5 vs. 5.9, *p* =0.03), healthcare costs (6.9 vs. 6.4, *p* = 0.02), and administrative work (6.8 vs. 6.1, *p* < 0.01). These professionals felt that TM would be less useful in reducing the frequency of in-person visits (6.0 vs. 6.4, *p* = 0.04) and were more concerned with the ease-of-use of the electronic devices (7.2 vs. 6.8, *p* =0.01) and with the need for incentives for the use of TM (6.6 vs. 6.1, *p* = 0.01). However, they were less concerned with the time needed for electronic visits (5.9 vs. 6.3, *p* = 0.03).

### Use of new technologies

#### Use of the Internet

Almost all the physicians (99.7%) stated that they regularly used Internet, and 93% had a mobile phone with an Internet connection. Table 4 shows the use of the Internet by professionals according to the device used, both personally and in the workplace. The devices used included: personal computers (99%), smartphone (93%) and tablets (48%). Social networks were used 12% more frequently by professionals with a tablet (confidence interval [CI] 95%: 6–17%). On the other hand, no relationship was found between having a tablet and using the Internet in the workplace.

**Table 4:** Use of the Internet according to the devices available

	PC (*n* = 757)	Smartphone (*n* = 709)	Tablet (*n* = 365)
Private Internet use			
Email (*n* = 755)	754 (99%)	706 (99%)	364 (99%)
Personal webpage (*n* = 91)	91 (12%)	88 (12%)	58 (16%)
Personal blog (*n* = 57)	57 (8%)	56 (8%)	41 (11%)
Use of non-medical apps (*n* = 685)	685 (91%)	663 (94%)	343 (94%)
Use of medical apps (*n* = 454)	452 (60%)	424 (60%)	216 (60%)
Social networks^a^ (*n* = 465)	465 (61%)	450 (64%)	267 (73%)
- Facebook^a^ (*n* = 424)	424 (60%)	412 (58%)	242 (66%)
- Twitter^a^ (*n* = 220)	220 (29%)	215 (30%)	139 (38%)
- Linkedin^a^ (*n* = 189)	189 (25%)	184 (26%)	119 (33%)
- Instagram^a^ (*n* = 115)	115 (15%)	115 (16%)	75 (21%)
Internet use in the workplace			
Internet use in the workplace (*n* = 742)	741 (98%)	694 (98%)	357 (98%)
Communication with professionals (*n* = 695)	694 (92%)	650 (92%)	336 (92%)
Communication with patients (*n* = 346)	346 (46%)	331 (47%)	184 (51%)
Recommendation of healthcare websites (*n* = 383)	383 (51%)	363 (51%)	194 (53%)
Recommendation of medical apps (*n* = 99)	99 (13%)	97 (14%)	51 (14%)
Patients consult healthcare websites (*n* = 330)	329 (44%)	313 (44%)	158 (43%)

^a^ *p* < 0.05 differences between users that use the Internet with a PC or smartphone versus a tablet using the Chi-squared test

The mean score of the importance of the Internet in the workplace was 8.2 points (SD 1.8). The importance of the Internet according to the professional uses of it is shown in Table 5. This score was higher among: users of the Internet in the workplace, professionals using the Internet to communicate with their patients and with other physicians that professionals not using it to these ends, those recommending health-related websites to their patients, and those with experience with telemedicine.

**Table 5:** Importance of the Internet according to profesional uses of it

Perception of the importance of the Internet in the workplace *n* = 760		Yes	No
	Use of the Internet in the workplace^a^	8.2 ± 1.7 (*n* = 742)	5.5 ± 2.9 (*n* = 17)
	Use of the Internet to communicate with other professionals^a^	8.3 ± 1.7 (*n* = 695)	7.1 ± 2.4 (*n* = 65)
	Use of the Internet to communicate with patients^a^	8.5 ± 1.6 (*n* = 346)	7.9 ± 1.9 (*n* = 412)
	Recommendation of health webpages to patients^a^	8.4 ± 1.6 (*n* = 383)	8.0 ± 1.9 (*n* = 377)
	Recommendation of medical apps to patients	8.4 ± 1.6 (*n* = 99)	8.1 ± 1.8 (*n* = 661)
	Patients’ questions on health information they have found online	8.2 ± 1.8 (*n* = 330)	8.1 ± 1.7 (*n* = 430)
	Experience with telemedicine^a^	8.4 ± 1.6 (*n* = 226)	8.1 ± 1.8 (*n* = 534)
Importance of the Internet in the workplace		Total *8.2* ± *1.8*	

Variables are scored on a scale of 1–10 (from least to greatest)

^a^ *p* < 0.05 differences between physicians using the Internet in different situations and physicians not using it using the T-Student Test

Table 6 shows the use of the Internet among physicians working in the public and the private sectors, with differences being observed in the communication with patients, with other professionals, and in the recommendation of websites between the two sectors.

**Table 6:** Internet use in the workplace according to sector

Internet use in the workplace			
	Public (*n* = 425)	Private (*n* = 142)	Both (*n* = 188)
Internet use in the workplace (*n* = 738)	417 (98%)	138 (97%)	183 (97%)
Communication with professionals^a^ (*n* = 690)	398 (94%)	123 (87%)	169 (90%)
Communication with patients^a^ (*n* = 343)	186 (44%)	78 (55%)	79 (42%)
Recommendation of health websites^a^ (*n* = 382)	235 (55%)	65 (46%)	82 (44%)
Recommendation of medical apps (*n* = 99)	50 (12%)	22 (16%)	27 (14%)
Patients consult health websites (*n* = 330)	195 (46%)	54 (38%)	81 (43%)

ª*p* < 0.05 difference between professionals working in public and private health according to the Chi-square test

#### How professionals feel that the Internet influences their patients

The mean score of the influence of the Internet on the health of the patients was 5.8 (SD 2.0), significantly differing according to whether the physician had a profile on a social network [6.0 (SD 2.0) vs. 5.5 (SD 1.9) *p* < 0.01].

## Discussion

According to a previous hypothesis by our group [19], physicians considered TM to be useful, especially those with previous experience with this technology. The attitude of healthcare professionals towards TM is a facilitating factor for the implementation of this type of project. Physicians favour the incorporation of technology into their daily lives provided that these innovations are useful [21].

Similar to previous studies [22], the results of our study conclude that having participated in a TM project is one of the factors that most influences physicians’ opinions of TM, resulting in a more positive view. In addition, although these professionals do not perceive more needs or difficulties than those who are not familiar with TM, they do perceive greater benefits with its use. It was of note that they considered that TM would improve the quality of clinical practice, patient health, and the professional workload. Nevertheless, they did demand projects with adequate funding. This can be explained in that the efficiency of some of these programs is not clear [23, 24]. Indeed, many pilot programs have not been implemented because of the lack of a economic feasibility plan associated with the study [25]. The ease-of-use of the electronic devices was of particular concern as was the need for incentives to use the technology. Professionals also demanded adequate technological teams and specific training in order to improve their technological skills, as has been reported by other groups [26, 27]. The main threat was considered to be the patients’ preference for in-person visits perhaps in concordance with the classical view of the physician-patient relationship.

The results of our study demonstrate the different view of the implementers, whose priorities differ from those of clinicians which are more related to efficiency. This is important for the implementation of TM since it is the implementers who decide as to the economic feasibility and project funding and how the project will be implemented. In order to generalize the use of TM the view of the implementers must be combined with that of the professionals taking into account the needs of the clinicians and their participation in the decision-making progress [28, 29]. Several factors may facilitate the implementation of technology in healthcare systems including the establishment of times during the workday to attend virtual visits and thereby avoiding an excessive workload, as well as the provision of incentives to professionals and improving the disposition of the health organization towards change [30, 31].

Professionals working in the private healthcare consider that TM will provide greater benefits, provided that the system is easy to use by their patients and incentives for its use are available. To this end, it is necessary to address the payment method for electronic visits in healthcare systems, since this factor may limit generalized use of TM.

The disassociation between professional and personal use of the Internet was also of note. That is, the profile of an Internet user did not condition how they used the Internet in the workplace or their opinion of TM. This is surprising, since according to the classical study by Rogers [32], and confirmed by Zanaboni in a study on the use of technology in health [33], the type of user is usually described according to at what time on the S-shaped logistic growth curve the user adopts the technology. Nevertheless, our findings suggest that it should be taken into account that a single user may have two roles, depending on whether we study their private or professional profile.

This disassociation between the personal and professional use of new technologies might initially seem paradoxical, but it may demonstrate the degree to which technology has entered into the private life of professionals. Nevertheless, there has been no change in the classical physician-patient relationship. That is, professionals have begun to see their world as being 2.0, but they still need to modify the way they work with their patients, and incorporate new technological tools [34]. Therefore, although professionals have adopted Internet for their personal use and consider it very important for their work, they do not feel that the health of their patients is affected by the use or not of the Internet to seek information related to healthcare.

With regard to the professional use of the Internet, although electronic communication between professionals does take place, it is not used to keep in contact with patients. On the other hand, physicians use very few medical applications compared with their use of other apps, and consequently, do not recommend these tools to their patients. There is little evidence of why this occurs, but it is likely that the lack of scientific evidence demonstrating their use [35] plays an important role in whether or not health-related apps are prescribed. The market is flooded with a rapid turnover of health applications which, in turn, quickly become obsolete, thereby obliging physicians to constantly update their knowledge. In addition, in our setting there are no institutional systems that fulfil the legal requirements for safety, nor is there any scientific entity that evaluates these tools and presents them in a simple way to professionals such as for example, for clinical histories in order to recommend their use to their patients. Although some progress has been made to this effect, there is still a long ways to go [36].

In relation to the personal use of the Internet, having a tablet is related to having a 2.0 attitude; that is, more intensive use of social networks. On the other hand, it is of note that having a smartphone does not determine how the Internet is used, probably because of their generalized use among the population, with most professionals having access to a telephone with a connection to the Internet. Indeed, we found a relationship between having a tablet and making greater use of the Internet and been more likely to use new technologies in the clinical practice.

Of note was the finding that no significant differences were observed in the Internet user profiles according to sex or age. Nevertheless, two profiles showed less intensive use of the Internet. The first profile was that of younger professionals without smartphones, who presented a less positive attitude towards e-health which may correspond with a philosophy that rejects the use of technology. The second profile included physicians over the age of 50 years who do not have tablets likely because the use of smartphones has become generalized in this age group and because of the digital divide [37].

Professionals working in private healthcare communicate more with their patients over the Internet, probably in order to provide more services. On the other hand, this communication is less frequent than that of professionals in the public health system in which an internal network facilitates communication.

In summary, physicians are open to the introduction of technology and consider it useful in daily practice. However, they feel that it should be implemented according to criteria that guarantee the quality of clinical care and adequate management of the technology to ensure its continuity On the other hand, these professionals have incorporated a 2.0 philosophy into their personal but not into their professional lives in that they do not take full advantage of what the Internet has to offer to them and to their patients.

### Limitations

The main methodological limitation of the present study is that the data were collected using a single questionnaire. In addition, the results are based on the opinions and the perceptions of the respondents and not necessarily on an objective reality.

Another limitation is that this was a cross sectional study, and thus, did not take into account possible changes in opinion over time. It’s particularly noteworthy to report the difficulty of accessing patients and physicians complying with the Data Protection Spanish law, which does not allow direct access. To comply with this law, professionals were contacted through the Health 2.0 Section of the Barcelona Medical Association. The institution has prestige among physicians which allowed us to have an acceptable participation in the study. Finally, we chose to use a questionnaire of our own design, designed and validated in a previous study of our team. This questionnaire sought to reflect doctors' perceptions of e-health, although without pretending to be exhaustive.

## Conclusions

Physicians believe in the usefulness of TM. Previous participation in a TM project is the factor that most influenced their opinion, leading to a greater consideration of it benefits rather than possible difficulties and shortcomings. Knowledge of the possibilities provided by technology helps in its application and optimization of the possible benefits that can be obtained from its use as well as having a more realistic outlook of the difficulties and needs involved.

While physicians have incorporated the 2.0 technology and philosophy into their private lives they have yet not done so in their daily work, and this is related to the use they make of it in the workplace. Healthcare systems and implementers must facilitate the use of technology by physicians at their professional lives and they have to analysed their needs in order to facilitate the generalized use of technology in healthcare.

The 2.0 philosophy has been incorporated into many areas of our lives, but healthcare systems still have a long way to go in order to incorporate this new way of understandingthe relationship between the patient, their health and their disease. The classical physician-patient relationship needs to evolve. Only in doing so will health professionals feel comfortable incorporating technology into how they interact with patients.

## Additional files

Additional file 1: Table S1. Sections and variables included the questionnaire. List of sections and variables included in the questionnaire. (DOCX 17 kb)

Additional file 2: Table S2. Opinion on telemedicine according to age and devices available. Opinion on telemedicine according to age and devices available. (DOC 154 kb)

## Abbreviations

CI: Confidence interval
PC: Personal computer
SD: standard desviation
TM: telemedicine
