# Supplementary material for: Implementing technology in healthcare: insights from physicians
Source: BMC Med Inform Decis Mak. 2017 Jun 27;17:92. doi: 10.1186/s12911-017-0489-2 (PMC5488364; doi:10.1186/s12911-017-0489-2)
Supplement: Supplementary file 1 — Sections and variables included the questionnaire. List of sections and variables included in the questionnaire. (DOCX 17 kb) [file 12911_2017_489_MOESM1_ESM.docx]

Additional file 1: Table S1

Sections and variables included the questionnaire.

| **Section I: Needs for the implementation of telemedicine** |
| --- |
| I.1. Patients’ preference for for in-person visits |
| I.2. Professionals’ preference for in-person visits |
| I.3. Patients’ technological skills |
| I.4. Professionals’ technological skills |
| I.5. Time needed for each patient |
| I.6. Appropiate electronic devices |
| I.7. Project funding |
| **Section II: Opportunities provided by telemedicine** |
| II.1. Quality of clinical practice |
| II.2. Patient health |
| II.3. Therapeutic compliance |
| II.4. Frequency of in-person visits |
| II.5. Professional workload |
| II.6. Healthcare costs |
| II.7. Administrative work |
| **Section III: Difficulties in implementing telemedicine** |
| III.1. Safety and confidentiality of information |
| III.2. Easy-of-use of electronic device |
| III.3. Record of professionals’ performance |
| III.4. Need for specific training |
| III.5. Technical difficulties in the use of new technology |
| III.6. Time needed for electronic visits |
| III.7. Presence of iincentives for the use of telemedicine |
| **Sección IV: Opinion on e-health** |
| IV.1. Influence of consulting medical information online on health |
| IV.2. Importance of the Internet in the workplace |
| IV.3. Usefulness of telemedicine |
| **Section V: Professional uses of the Internet** |
| V.1. Use of the Internet in the workplace |
| V.2. Use of the Internet to communicate with other professionals |
| V.3. Use of the Internet to communicate with patients |
| V.4. Recommendation of health webpages to patients |
| V.5. Recommendation of medical apps to patients |
| V.6. Patients’ questions on health information they have found online |
| V.7. Experience with telemedicine |
| **Section VI: Personal use of the Internet** |
| VI.1. Electronic devices available |
| VI.2. Email |
| VI.3.Personal webpage |
| VI.4. Personal blog |
| VI.5. Use of non-medical apps |
| VI.6. Use of medical apps |
| VI.7. Use of social networks |
| **Section VII: Sociodemographic information** |
| VII.1. Age |
| VII.2. Sex |
| VII.3. Residence |
| VII.4. Specialty |
| VII.5. Sector |
| VII.6. Place of work |
| VII.7. Position |
| **Section VIII: Comments** |
