# Supplementary material for: Implementing technology in healthcare: insights from physicians
Source: BMC Med Inform Decis Mak. 2017 Jun 27;17:92. doi: 10.1186/s12911-017-0489-2 (PMC5488364; doi:10.1186/s12911-017-0489-2)
Supplement: Supplementary file 2 — Opinion on telemedicine according to age and devices available. Opinion on telemedicine according to age and devices available. (DOC 154 kb) [file 12911_2017_489_MOESM2_ESM.doc]

**Additional file 2: Table S2**

**Opinion on Telemedicine of patients under 40 years old according to devices available**

|  |  | **PC** | | **Smartphone** | | | **Tablet** | |
| --- | --- | --- | --- | --- | --- | --- | --- | --- |
|  |  | **Yes (n=285)** | **Yes (n=280)** | | **No (n=5)** | **Yes (n=148)** | | **No (n=137)** |
| **Section I: Needs for the implementation of telemedicine** | I.1. Preference of patients for in-person visits | 6.4 + 1.9 | 6.4 + 1.9 | | 6.4 + 0.9 | 6.6 + 2.0 | | 6.3 + 2.0 |
|  | I.2. Preference of healthcare professionals for in-person visits | 6.2 + 1.9 | 6.2+ 2.0 | | 5.8 + 1.3 | 6.4 + 1.9 | | 6.0 + 2.0 |
|  | I.3. Patients’ technological skills | 7.0 + 1.8 | 7.0 + 1.8 | | 6.2 + 1.5 | 7.2 + 1.8 | | 6.9 + 1.8 |
|  | I.4. Professionals’ technological skills | 6.8 + 1.8 | 6.8 + 1.9 | | 6.4 + 1.1 | 6.9 + 1.8 | | 6.7 + 1.8 |
|  | I.5. Time needed for each patient | 6.1 + 1.9 | 6.1 + 1.8 | | 6.4 + 1.7 | 6.2 + 1.8 | | 6.0 + 1.9 |
|  | I.6. Adequate technological team | 7.7 + 1.9 | 7.7 + 1.9 | | 7.6 + 0.9 | 7.7 + 1.8 | | 7.7 + 1.9 |
|  | I.7. Project funding | 8.0 + 1.7 | 8.0 + 1.8 | | 8.2 + 1.1 | 8.0 + 1.7 | | 8.0 + 1.8 |
| **Section II: Opportunities provided by telemedicine** | II.1. Quality of clinical practice | 6.5 + 1.7 | 6.5 + 1.7 | | 5.6 + 1.7 | 6.5 + 1.6 | | 6.5 + 1.8 |
|  | II.2. Patient health^a^ | 6.5 + 1.5 | 6.5 + 1.5 | | 4.6 + 2.3 | 6.6 + 1.5 | | 6.4 + 1.6 |
|  | II.3. Therapeutic compliance^a^ | 6.7 + 1.7 | 6.7 + 1.7 | | 4.8 +2.8 | 6.8 + 1.7 | | 6.6 + 1.7 |
|  | II.4. Frequency of in-person visits | 6.5 + 1.9 | 6.6 + 1.9 | | 5.8 + 1.9 | 6.6 + 1.8 | | 6.5 + 2.0 |
|  | II.5. Professional workload | 5.9 + 2.1 | 5.9 + 2.1 | | 4.8 + 1.9 | 6.0 + 2.1 | | 5.8 + 2.1 |
|  | II.6. Healthcare costs | 6.7 + 1.6 | 6.7 + 1.6 | | 6.4 + 1.5 | 6.9 + 1.4 | | 6.6 + 1.8 |
|  | II.7. Administrative work | 6.1 + 2.1 | 6.1 + 2.1 | | 4.8 + 3.0 | 6.2 + 2.0 | | 6.0 + 2.3 |
| **Section III: Difficulties in implementing telemedicine** | III.1. Safety and confidentiality of information | 5.6 + 2.1 | 5.6 + 2.1 | | 6.8 + 2.1 | 5.6 + 2.2 | | 5.7 + 2.0 |
|  | III.2. Easy-of-use of electronic devices | 7.0 + 1.7 | 7.0 + 1.7 | | 6.6 + 0.9 | 6.9 + 1.8 | | 7.1 + 1.6 |
|  | III.3. Record of professionals’ performance | 6.7 + 1.8 | 6.7 + 1.8 | | 6.0 + 1.0 | 6.7 + 1.8 | | 6.8 + 1.9 |
|  | III.4. Need for specific training | 6.9 + 1.7 | 6.9 + 1.7 | | 6.0 + 1.6 | 6.7 + 1.8 | | 7.0 + 1.6 |
|  | III.5. Technical difficulties in the use of new technology | 6.2 + 1.8 | 6.2 + 1.8 | | 5.6 + 1.5 | 6.3 + 1.8 | | 6.1 + 1.8 |
|  | III.6. Time needed for electronic visit | 6.5 + 1.9 | 6.5 + 1.9 | | 6.6 + 1.5 | 6.6 + 1.9 | | 6.3 + 1.8 |
|  | III.7. Incentives for the use of telemedicine | 6.3 + 2.1 | 6.3 + 2.1 | | 5.8 + 0.8 | 6.2 + 2.1 | | 6.3 + 2.1 |
| **Section IV: Opinion on e-health** | IV.1. Influence of consulting medical information online on health | 6.0 + 1.9 | 6.0 + 1.9 | | 5.4 + 2.1 | 6.2 + 2.0 | | 5.8 + 1.9 |
|  | IV.2. Importance of the Internet in the workplace | 8.2 + 1.5 | 8.3 + 1.5 | | 8.6 + 1.9 | 8.3 + 1.5 | | 8.3 + 1.5 |
|  | IV.3. Usefulness of telemedicine^a^ | 7.4 + 1.6 | 7.4 + 1.6 | | 5.6 + 1.8 | 7.5 + 1.7 | | 7.3 + 1.6 |

Variables are scored on a scale of 1 to 10 (from least to greatest)

^a^p<0.05 difference between having or not having smartphone using the T-student test

^b^p<0.05 difference between having or not having tablet using the T-student test

**Opinion on Telemedicine of patients from 40-49 years old according to devices available**

|  |  | **PC** | | **Smartphone** | | | **Tablet** | |
| --- | --- | --- | --- | --- | --- | --- | --- | --- |
|  |  | **Yes (n=163)** | **Yes (n=152)** | | **No (n=11)** | **Yes (n=69)** | | **No (n=94)** |
| **Section I: Needs for the implementation of telemedicine** | I.1. Preference of patients for in-person visits | 6.8 +2.1 | 6.8 + 2.0 | | 6.6 + 2.4 | 7.1+ 2.1 | | 6.6 + 2.0 |
|  | I.2. Preference of healthcare professionals for in-person visits | 6.5 + 2.0 | 6.5 + 2.0 | | 6.9 + 2.0 | 6.8 + 1.8 | | 6.3 + 2.1 |
|  | I.3. Patients’ technological skills | 6.8 + 1.9 | 6.8 + 1.9 | | 6.9 + 2.1 | 6.9 + 1.9 | | 6.7 + 1.9 |
|  | I.4. Professionals’ technological skills | 6.6 + 2.0 | 6.6 + 2.0 | | 7.0 + 1.9 | 6.9 + 1.9 | | 6.4 + 2.0 |
|  | I.5. Time needed for each patient | 6.4 + 2.2 | 6.2 + 2.2 | | 7.3 + 2.3 | 6.4 + 2.1 | | 6.3 + 2.3 |
|  | I.6. Adequate technological team | 7.3 + 2.3 | 7.3 + 2.3 | | 7.8 + 2.4 | 7.5 + 2.3 | | 7.2 + 2.3 |
|  | I.7. Project funding | 7.5 + 2.3 | 7.5 + 2.3 | | 8.3 + 1.9 | 7.8 + 2.1 | | 7.3 + 2.4 |
| **Section II: Opportunities provided by telemedicine** | II.1. Quality of clinical practice | 6.6 + 1.9 | 6.7 + 1.9 | | 5.9 + 1.9 | 6.6 + 1.8 | | 6.7 + 2.0 |
|  | II.2. Patient health^a^ | 6.8 + 1.6 | 6.9 + 1.6 | | 5.6 + 1.7 | 6.8 + 1.6 | | 6.8 + 1.7 |
|  | II.3. Therapeutic compliance | 6.6 + 1.7 | 6.6 + 1.7 | | 6.1 +2.5 | 6.5 + 1.7 | | 6.6 + 1.8 |
|  | II.4. Frequency of in-person visits | 6.4 + 1.9 | 6.5 + 1.9 | | 5.6 + 2.0 | 6.7 + 2.1 | | 6.2 + 1.8 |
|  | II.5. Professional workload^b^ | 5.9 + 2.2 | 6.0 + 2.2 | | 5.1 + 2.6 | 6.5 + 2.2 | | 5.5 + 2.1 |
|  | II.6. Healthcare costs | 6.6 + 2.0 | 6.7 + 2.0 | | 5.6 + 2.3 | 6.9 + 2.0 | | 6.4 + 2.0 |
|  | II.7. Administrative work | 6.5 + 2.2 | 6.5 + 2.1 | | 5.6 + 2.9 | 6.6 + 2.4 | | 6.4 + 2.1 |
| **Section III: Difficulties in implementing telemedicine** | III.1. Safety and confidentiality of information | 5.3 + 2.4 | 5.4 + 2.4 | | 4.6 + 19 | 5.7 + 2.3 | | 5.1 + 2.4 |
|  | III.2. Easy-of-use of electronic devices | 6.6 + 2.0 | 6.7 + 1.9 | | 5.6 + 2.8 | 6.7 + 2.0 | | 6.4 + 2.0 |
|  | III.3. Record of professionals’ performance | 6.5 + 2.2 | 6.5 + 2.2 | | 5.7 + 2.2 | 6.7 + 2.1 | | 6.3 + 2.4 |
|  | III.4. Need for specific training^b^ | 6.9 + 2.2 | 6.9 + 2.1 | | 6.5 + 2.2 | 7.3 + 1.9 | | 6.6 + 2.4 |
|  | III.5. Technical difficulties in the use of new technology | 6.0 + 2.3 | 6.0 + 2.2 | | 5.8 + 2.8 | 6.2 + 2.2 | | 5.8 + 2.3 |
|  | III.6. Time needed for electronic visit | 6.2 + 2.5 | 6.2 + 2.5 | | 6.4 + 3.0 | 6.3 + 2.4 | | 6.2 + 2.5 |
|  | III.7. Incentives for the use of telemedicine | 6.0 + 2.7 | 5.9 + 2.7 | | 7.2 + 3.2 | 6.1 + 2.7 | | 6.0 + 2.8 |
| **Section IV: Opinion on e-health** | IV.1. Influence of consulting medical information online on health | 5.8 + 2.0 | 5.7 + 2.0 | | 5.9 + 2.2 | 6.0 + 1.8 | | 5.6 + 2.1 |
|  | IV.2. Importance of the Internet in the workplace | 8.0 + 2.0 | 7.9 + 2.0 | | 8.6 + 1.2 | 7.8 + 2.4 | | 8.0 + 1.7 |
|  | IV.3. Usefulness of telemedicine | 7.2 + 2.0 | 7.3 + 2.0 | | 6.7 + 2.0 | 7.4 + 2.0 | | 7.2 + 2.0 |

Variables are scored on a scale of 1 to 10 (from least to greatest)

^a^p<0.05 difference between having or not having smartphone using the T-student test

^b^p<0.05 difference between having or not having tablet using the T-student test

**Opinion on Telemedicine of patients over 50 years old according to devices available**

|  | |  | **PC** | | **Smartphone** | | | | **Tablet** | |
| --- | --- | --- | --- | --- | --- | --- | --- | --- | --- | --- |
|  |  |  | **Yes (n=309)** | **Yes (n=277)** | | | **No (n=35)** | **Yes (n=148)** | | **No (n=164)** |
| **Section I: Needs for the implementation of telemedicine** | | I.1. Preference of patients for in-person visits | 6.4 +2.3 | 6.4 + 2.4 | | | 6.6 + 2.1 | 6.3 + 2.4 | | 6.5 + 2.2 |
|  |  | I.2. Preference of healthcare professionals for in-person visits | 6.6 + 2.0 | 6.7 + 2.0 | | | 6.3 + 1.9 | 6.6 + 2.0 | | 6.6 + 2.0 |
|  |  | I.3. Patients’ technological skills^a^ | 6.3 + 2.0 | 6.4 + 2.0 | | | 5.8 + 2.0 | 6.4 + 2.1 | | 6.3 + 1.9 |
|  |  | I.4. Professionals’ technological skills | 7.1 + 1.9 | 7.2 + 1.8 | | | 6.3 + 2.0 | 7.2 + 1.9 | | 7.0 + 1.9 |
|  |  | I.5. Time needed for each patient | 6.3 + 2.0 | 6.3 + 2.0 | | | 6.2 + 2.1 | 6.5 + 2.0 | | 6.1 + 2.1 |
|  |  | I.6. Adequate technological team | 7.7 + 2.0 | 7.7 + 2.0 | | | 7.5 + 1.7 | 7.8 + 2.1 | | 7.5 + 1.9 |
|  |  | I.7. Project funding^b^ | 7.8 + 2.0 | 7.9 + 2.1 | | | 7.6 + 2.0 | 8.1 + 2.0 | | 7.6 + 2.1 |
| **Section II: Opportunities provided by telemedicine** | | II.1. Quality of clinical practice^b^ | 6.6 + 2.0 | 6.6 + 2.1 | | | 6.0 + 2.0 | 7.0 + 1.8 | | 6.2 + 2.1 |
|  |  | II.2. Patient health^b^ | 6.6 + 1.8 | 6.6 + 1.8 | | | 6.2 + 1.6 | 7.0 + 1.8 | | 6.2+ 1.8 |
|  |  | II.3. Therapeutic compliance^b^ | 6.6 + 1.9 | 6.7 + 2.0 | | | 6.1 + 1.8 | 7.0 + 2.0 | | 6.3 + 1.9 |
|  |  | II.4. Frequency of in-person visits | 6.1 + 2.0 | 6.1 + 2.0 | | | 6.0 + 1.8 | 6.1 + 2.0 | | 6.0 + 2.0 |
|  |  | II.5. Professional workload | 6.3 + 1.9 | 6.4 + 1.9 | | | 6.1 + 2.0 | 6.5 + 2.0 | | 6.2 +1.9 |
|  |  | II.6. Healthcare costs^b^ | 6.3 + 1.8 | 6.4 + 1.9 | | | 5.8 + 1.4 | 6.5 + 1.9 | | 6.1 + 1.8 |
|  |  | II.7. Administrative work^b^ | 6.4 + 2.1 | 6.5 + 2.1 | | | 6.1 + 1.7 | 6.7 + 2.1 | | 6.2 + 2.1 |
| **Section III: Difficulties in implementing telemedicine** | | III.1. Safety and confidentiality of information | 6.0 + 2.3 | 6.1 + 2.3 | | | 5.6 + 2.0 | 5.9 + 2.4 | | 6.0 + 2.3 |
|  |  | III.2. Easy-of-use of electronic devices^b^ | 7.0 + 1.9 | 7.1 + 1.9 | | | 6.6 + 2.0 | 7.3 + 1.9 | | 6.8 + 1.9 |
|  |  | III.3. Record of professionals’ performance^b^ | 6.9 + 2.1 | 7.0 + 2.1 | | | 6.5 + 1.9 | 7.2 + 2.1 | | 6.6 + 2.1 |
|  |  | III.4. Need for specific training | 7.6 + 1.8 | 7.6 +1.8 | | | 7.6 + 1.9 | 7.7 + 2.0 | | 7.5 + 1.7 |
|  |  | III.5. Technical difficulties in the use of new technology | 6.5 + 2.0 | 6.5 + 2.0 | | | 6.6 + 2.0 | 6.5 + 2.1 | | 6.5 + 1.9 |
|  |  | III.6. Time needed for electronic visit | 6.5 + 2.1 | 6.6 + 2.1 | | | 6.1 + 2.0 | 6.7 + 2.1 | | 6.4 + 2.0 |
|  |  | III.7. Incentives for the use of telemedicine^b^ | 6.3 + 2.3 | 6.4 + 2.4 | | | 5.7 + 2.0 | 6.7 + 2.3 | | 6.0 + 2.3 |
| **Section IV: Opinion on e-health** | | IV.1. Influence of consulting medical information online on health | 5.5 + 2.1 | 5.5 + 2.2 | | | 5.7 + 1.7 | 5.7 + 2.3 | | 5.4 + 2.0 |
|  |  | IV.2. Importance of the Internet in the workplace^b^ | 8.2 + 1.8 | 8.3 + 1.7 | | | 7.1 + 2.3 | 8.4 + 1.7 | | 7.9 + 1.9 |
|  |  | IV.3. Usefulness of telemedicine^b^ | 7.6 + 1.9 | 7.6 + 1.9 | | | 7.3 + 1.8 | 7.8 + 1.7 | | 7.3 + 1.9 |
|  |  | | | | |  | | | | |

Variables are scored on a scale of 1 to 10 (from least to greatest)

^a^p<0.05 difference between having or not having smartphone using the T-student test

^b^p<0.05 difference between having or not having tablet using the T-student test
